# Correlations Among Glycemia and Glucose Variability, Thyroid Hormones, and Albuminuria in Patients With Type 2 Diabetes Mellitus

**DOI:** 10.1002/kjm2.70221

**Published:** 2026-04-22

**Authors:** Na Luo, Shang‐Yong Feng, Hui Chen, Dun‐Min She, Yan Liu, Ying Li, Cai‐Feng Yan, Zhen‐Wen Zhang, Jing Chen, Xiao‐Li Cai, Gao‐Yu Zhao, Hui Yu, Yan Wang

**Affiliations:** ^1^ Department of Endocrinology Northern Jiangsu People's Hospital Yangzhou Jiangsu China; ^2^ Department of Endocrinology Hospital of Yangzhou HongQuan Yangzhou Jiangsu China; ^3^ Department of Endocrinology Chongqing Tongnan District People's Hospital Chongqing Sichuan China

**Keywords:** albuminuria, continuous glucose monitoring system, glucose variability, thyroid hormone, type 2 diabetes mellitus

## Abstract

Hyperglycemia, glucose fluctuations, and thyroid dysfunction contribute to the progression of diabetic nephropathy. This study aimed to investigate the associations of glycemia, glucose variability, and thyroid hormones with albuminuria in patients with type 2 diabetes mellitus (T2DM). In total, 451 T2DM patients were included. The data of continuous glucose monitoring system (CGMS)‐generated indices, thyroid hormones, and the ratios of urine creatinine to urine microalbumin (URCA) were collected. There were 152 patients with normal URCA (< 30 mg/g), 230 patients with microalbuminuria (30 mg/g ≤ UACR < 300 mg/g), and 69 patients with macroalbuminuria (UACR ≥ 300 mg/g). Free triiodothyronine (FT3) was negatively correlated with hemoglobin A1C (HbA1C), glucose, and glucose variability in T2DM patients (most *p* < 0.05). HbA1C (*p* = 0.002), the standard deviation (SD) of glucose (*p* < 0.001), the coefficient of variation (CV) of glucose (*p* < 0.001), the time below range (TBR) (*p* = 0.002), and the mean amplitude of glycemic excursions (MAGE) (*p* = 0.033) were positively associated with albuminuria. Moreover, FT3 was negatively associated with macroalbuminuria (*p* = 0.003). According to multivariate logistic regression analyses, HbA1C, SD, and CV of glucose, the TBR, and the MAGE were independently associated with a greater risk of albuminuria after adjustment for demographics, duration of T2DM, biochemical indices, and medications for T2DM and hypertension (all *p* < 0.05). Moreover, the associations of HbA1C, SD, and CV of glucose, TBR, and MAGE with albuminuria were partially mediated by FT3, FT3, and TSH, but the weight of mediation was low. In conclusion, glycemia and glucose variability derived from the CGMS are correlated with a lower level of FT3, and they are positively associated with albuminuria in patients with T2DM.

## 1 Introduction

Diabetic nephropathy is a common microvascular complication of diabetes mellitus (DM), which is clinically manifested as an increase in albuminuria and a decrease in the glomerular filtration rate (GFR) [1, 2]. Diabetic nephropathy is a vital cause of end‐stage renal disease, which severely hampers the quality of life and life expectancy of patients with DM [3, 4, 5]. Therefore, implementing proper management to reduce the incidence of diabetic nephropathy is highly important.

The pathogenesis and progression of diabetic nephropathy are associated with multiple factors. Hyperglycemia is considered a vital factor that contributes to diabetic nephropathy and induces oxidative stress, inflammation, and apoptosis in renal tissues [6, 7, 8]. Consequently, adequate and sustained control of blood glucose is critical to prevent diabetic nephropathy. The continuous glucose monitoring system (CGMS) is an emerging technology that reflects the level and variability of blood glucose and has been applied for monitoring the control of hyperglycemia [9, 10]. To date, the associations of indices generated by CGMS with diabetic nephropathy have been reported in only a few studies, and inconsistencies exist among them [11, 12]. For example, one study reported that indices reflecting the variation in blood glucose from CGMS were associated with the severity of albuminuria [11], whereas another study reported the opposite findings [12]. Therefore, this issue needs to be further explored.

Thyroid hormones are crucial for regulating the metabolism and activity of multiple organs [13]. Previous studies have reported the associations of thyroid hormones with albuminuria in patients with DM [14, 15, 16]. Moreover, it has also been reported that glycemic variability is associated with thyroid hormones in patients with type 2 DM (T2DM) [17]. These studies led to the question of whether the association of glycemic variability with albuminuria was mediated by thyroid hormones. However, no study has explored this issue.

This study aimed to explore the associations of indices generated by the CGMS and thyroid hormones with albuminuria, as well as the correlations between indices generated by the CGMS and thyroid hormones in patients with T2DM. In addition, the effects of thyroid hormones on the associations between CGMS indices and albuminuria were also investigated.

## 2 Methods

### 2.1 Patients

In this retrospective study, T2DM patients who wore CGMS (iPro 2, Medtronic Inc.) equipment from April 2018 to December 2020 were consecutively included. In our hospital, CGMS is primarily prescribed for hospitalized patients with inadequate glycemic control or marked glycemic variability, particularly those with suspected asymptomatic hypoglycemia, for whom there is a need to evaluate specific glycemic patterns (e.g., nocturnal or postprandial glucose excursions) or to guide therapeutic adjustments such as the initiation or intensification of insulin therapy. The inclusion criteria were as follows: (a) diagnosed with T2DM according to the 1999 WHO diagnostic criteria; (b) aged over 18 years; (c) wore CGMS equipment for at least 3 days; and (d) had available clinical data for study analysis, including demographics, disease‐related indices, biochemical indices, CGMS‐derived indices, and thyroid function indices (described in detail below). The exclusion criteria were (a) a diagnosis of other forms of diabetes; (b) diabetic ketoacidosis or hyperosmolar coma; (c) severe liver impairment; (d) a previous history of thyroid surgery or treatment for thyroid disease; (e) the use of medications known to affect thyroid function (e.g., high‐dose glucocorticoids and amiodarone); and (f) pregnancy or breastfeeding. The study received approval from the Ethics Committee of Northern Jiangsu People's Hospital (approval number: 2020ky‐085; date of approval: Jan. 8th, 2021).

### 2.2 Data Collection

The data of T2DM patients were sorted from medical records, which included (a) demographic data, such as age, sex, height, weight, smoking status, and history of hypertension; (b) disease‐related indices, such as duration of T2DM; and (c) biochemical indices, such as blood pressure, cholesterol (CHO), triglyceride (TG), blood urea nitrogen (BUN), creatinine (Cr), and estimated GFR (eGFR). The eGFR was calculated via the following formula: 175 × [Serum Cr]^−1.234^ × [Age]^−0.179^ × Sex factor (male: 1, female: 0.79). In addition, the CGMS‐derived indices and thyroid function indices were also collected, which included hemoglobin A1c (HbA1C), the mean glucose level, the standard deviation (SD) of glucose, the time below range (TBR, ≤ 3.9 mmol/L), the time in range (TIR, 3.9–10.0 mmol/L), the time above range (TAR, ≥ 10.0 mmol/L), the coefficient of variation (CV), the mean amplitude of glycemic excursions (MAGE), the free triiodothyronine (FT3), the free thyroxine (FT4), and the thyroid stimulating hormone (TSH). Medication information for glucose and blood pressure control was also collected.

### 2.3 Grouping

The ratios of urine creatinine to urine microalbumin (UACR) of patients were also collected. UACR was measured in a single spot urine sample. Specifically, a midstream sample from the first morning urine on the second day of hospitalization was collected and urinary albumin and creatinine concentrations were determined via an automated analyzer (Hitachi 7600, Hitachi Ltd., Japan). Patients were classified into (a) the normal group, < 30 mg/g; (b) the microalbuminuria group, UACR ≥ 30 mg/g and < 300 mg/g; and (c) the macroalbuminuria group, UACR ≥ 300 mg/g.

### 2.4 Statistics

SPSS v.26.0 (IBM, USA) was used to analyze the clinical data. Comparisons among three groups were conducted via the Kruskal–Wallis H rank sum test, one‐way analysis of variance, or the *χ* ^2^ test. Correlations between glycemic and thyroid function indices were determined via Spearman's rank correlation test. The factors related to the risk of albuminuria or macroalbuminuria were analyzed via univariate logistic regression analysis. The CGMS‐derived indices or thyroid function indices with *p* < 0.05 in univariate analyses for the risk of albuminuria or macroalbuminuria were further included in multivariate logistic regression analysis to explore their independent effects after adjustment for other indices via 4 different models. In detail, demographics were adjusted by Model 1; demographics and disease‐related indices were adjusted by Model 2; demographics, disease‐related indices, and biochemical indices were adjusted by Model 3; and demographics, disease‐related indices, biochemical indices, and medication information were adjusted by Model 4. Receiver operating characteristic (ROC) curve analysis was performed to determine the optimal cutoff values for discriminating the risk of albuminuria or macroalbuminuria, with the optimal threshold defined by the maximum Youden index. Missing data were handled via complete‐case analysis. Mediation analysis based on regression models was used to assess the potential mediating role of thyroid hormone levels in the association between glycemic status and the risk of albuminuria. *p* < 0.05 indicated statistical significance.

## 3 Results

### 3.1 Basic Characteristics

A total of 679 patients were screened, 228 of whom were excluded, leaving 451 patients included in this study. The mean age of the T2DM patients was 58.1 ± 14.7 years; 157 (34.8%) were females, and 294 (65.2%) were males. The 451 patients with T2DM were divided into a normal group (*n* = 152), a microalbuminuria group (*n* = 230), and a macroalbuminuria group (*n* = 69) according to the UACR. Comparisons among the normal, microalbuminuria, and macroalbuminuria groups revealed that age, sex, diastolic blood pressure (DBP), smoking status, high‐density lipoprotein cholesterol (HDL‐C), and low‐density lipoprotein cholesterol (LDL‐C) were similar among the three groups (all *p* > 0.05). However, body mass index (BMI), systolic blood pressure (SBP), history of hypertension, duration of T2DM, CHO, TG, uric acid, BUN, Cr, eGFR, URCA, use of sulfonylureas, glucagon‐like peptide‐1 (GLP‐1) inhibitor, calcium channel blockers (CCBs), and angiotensin II receptor blockers (ARBs) were different among the three groups (all *p* < 0.05) (Table 1).

**TABLE 1:** Clinical characteristics.

Items	Total T2DM patients (*N* = 451)	Normal (*n* = 152)	Microalbuminuria (*n* = 230)	Macroalbuminuria (*n* = 69)	*p*
Demographics					
Age (years), mean ± SD	58.1 ± 14.7	58.2 ± 14.3	57.6 ± 15.6	59.5 ± 12.5	0.635
Sex, no. (%)					0.080
Female	157 (34.8)	54 (35.5)	87 (37.8)	16 (23.2)	
Male	294 (65.2)	98 (64.5)	143 (62.2)	53 (76.8)	
BMI (kg/m^2^), mean ± SD	24.9 ± 3.7	24.3 ± 3.2	24.9 ± 3.9	26.1 ± 3.8	0.003
SBP (mmHg), mean ± SD	136.5 ± 17.7	134.0 ± 14.7	135.4 ± 17.7	145.7 ± 21.1	< 0.001
DBP (mmHg), mean ± SD	80.6 ± 10.9	79.4 ± 8.5	80.8 ± 11.6	83.1 ± 12.5	0.065
Smoke, no. (%)					0.195
No	316 (71.7)	109 (75.2)	164 (71.9)	43 (63.2)	
Yes	125 (28.3)	36 (24.8)	64 (28.1)	25 (36.8)	
History of hypertension, no. (%)					< 0.001
No	206 (45.8)	84 (55.6)	107 (46.5)	15 (21.7)	
Yes	244 (54.2)	67 (44.4)	123 (53.5)	54 (78.3)	
Disease‐related indices					
Duration of T2DM (years), median (IQR)	10.0 (4.0–16.0)	10.0 (4.8–17.0)	8.0 (2.0–15.0)	10.0 (8.0–20.0)	< 0.001
Biochemical indices					
CHO (mmol/L), median (IQR)	4.2 (3.5–5.0)	4.1 (3.5–4.7)	4.2 (3.5–5.0)	4.6 (3.5–6.0)	0.023
HDL‐C (mmol/L), median (IQR)	1.0 (0.8–1.2)	1.0 (0.9–1.2)	0.9 (0.8–1.1)	0.9 (0.8–1.1)	0.082
LDL‐C (mmol/L), median (IQR)	2.4 (1.8–3.0)	2.3 (1.9–2.9)	2.4 (1.8–3.0)	2.5 (1.9–3.4)	0.246
TG (mmol/L), median (IQR)	1.7 (1.2–2.6)	1.5 (1.1–2.2)	1.8 (1.2–2.8)	1.9 (1.4–2.9)	0.002
Uric acid (μmol/L), median (IQR)	312.0 (243.9–384.8)	290.0 (227.0–362.0)	314.5 (249.0–389.3)	360.0 (296.5–431.5)	< 0.001
BUN (mmol/L), median (IQR)	5.7 (4.7–7.2)	5.4 (4.5–6.7)	5.7 (4.7–7.1)	6.7 (5.8–9.6)	< 0.001
Cr (μmol/L), median (IQR)	77.0 (67.0–96.3)	74.0 (64.0–84.0)	76.0 (66.0–92.0)	100.0 (81.0–144.0)	< 0.001
eGFR (mL/min/1.73 m^2^), median (IQR)	88.2 (65.3–106.0)	93.0 (75.2–111.7)	91.1 (68.2–109.3)	64.4 (44.7–79.6)	< 0.001
UACR (mg/g), median (IQR)	49.9 (19.8–127.7)	14.0 (7.4–20.4)	70.3 (43.6–103.5)	701.2 (521.4–1484.4)	< 0.001
Medication information					
Insulin use, *n* (%)	364 (80.7)	124 (81.6)	181 (78.7)	59 (85.5)	0.429
Sulfonylureas use, *n* (%)	75 (16.6)	21 (13.8)	48 (20.9)	6 (8.7)	0.030
Benzoic acid derivative use, *n* (%)	54 (12.0)	25 (16.4)	24 (10.4)	5 (7.2)	0.088
Biguanide use, *n* (%)	296 (65.6)	101 (66.4)	158 (68.7)	37 (53.6)	0.067
DPP‐4 inhibitor use, *n* (%)	117 (25.9)	40 (26.3)	60 (26.1)	17 (24.6)	0.963
Glycosidase inhibitor use, *n* (%)	176 (39.0)	62 (40.8)	86 (37.4)	28 (40.6)	0.768
TZD use, *n* (%)	5 (1.1)	2 (1.3)	2 (0.9)	1 (1.4)	0.711
GLP‐1 inhibitor use, *n* (%)	32 (7.1)	6 (3.9)	16 (7.0)	10 (14.5)	0.027
SGLT‐2 inhibitor use, *n* (%)	26 (5.8)	12 (7.9)	11 (4.8)	3 (4.3)	0.400
β‐receptor blocker use, *n* (%)	36 (8.0)	11 (7.2)	19 (8.3)	6 (8.7)	0.911
CCB use, *n* (%)	111 (24.6)	26 (17.1)	54 (23.5)	31 (44.9)	< 0.001
ACE inhibitor use, *n* (%)	14 (3.1)	5 (3.3)	7 (3.0)	2 (2.9)	1.000
ARB use, *n* (%)	142 (31.5)	34 (22.4)	68 (29.6)	40 (58.0)	< 0.001
MRA use, *n* (%)	11 (2.4)	2 (1.3)	6 (2.6)	3 (4.3)	0.311
Diuretic use, *n* (%)	24 (5.3)	5 (3.3)	12 (5.2)	7 (10.1)	0.116
α‐receptor blocker use, *n* (%)	4 (0.9)	2 (1.3)	1 (0.4)	1 (1.4)	0.481

Abbreviations: ACE, angiotensin‐converting enzyme; ARB, angiotensin II receptor blocker; BMI, body mass index; BUN, blood urea nitrogen; CCB, calcium channel blocker; CHO, cholesterol; Cr, creatinine; DBP, diastolic blood pressure; DPP‐4, dipeptidyl peptidase‐4; eGFR, estimated glomerular‐filtration rate; GLP‐1, glucagon‐like peptide‐1; HDL‐C, high‐density lipoprotein cholesterol; IQR, interquartile range; LDL‐C, low‐density lipoprotein cholesterol; MRA, mineralocorticoid receptor antagonist; SBP, systolic blood pressure; SD, standard deviation; SGLT‐2, sodium–glucose cotransporter 2; T2DM, type 2 diabetes mellitus; TG, triglyceride; TZD, thiazolidinedione; UACR, ratio of urine creatinine to urine microalbumin.

### 3.2 Comparison of CGMS‐Derived Indices and Thyroid Function Indices Among Groups

The detailed CGMS‐derived indices and thyroid function indices of all the T2DM patients and each group are presented in Table 2. By comparison analyses, HbA1C (*p* = 0.008), the SD of glucose (*p* < 0.001), the TBR (*p* = 0.002), the CV of glucose (*p* < 0.001), and FT3 (*p* < 0.001) were different among the normal, microalbuminuria, and macroalbuminuria groups. However, the means of glucose, TIR, TAR, MAGE, FT4, and TSH did not differ among the three groups (all *p* > 0.05).

**TABLE 2:** CGMS‐derived indices and thyroid function indices.

Items	Total T2DM patients (*N* = 451)	Normal (*n* = 152)	Microalbuminuria (*n* = 230)	Macroalbuminuria (*n* = 69)	*p*
HbA1C (%), mean ± SD	9.9 ± 2.4	9.4 ± 2.2	10.1 ± 2.3	10.0 ± 2.6	0.008
Mean of glucose (mmol/L), mean ± SD	9.3 ± 1.9	9.2 ± 2.0	9.4 ± 2.0	9.5 ± 1.5	0.509
SD of glucose (mmol/L), mean ± SD	2.3 ± 0.9	2.0 ± 0.7	2.4 ± 0.9	2.4 ± 0.9	< 0.001
TBR (%), mean ± SD	0.8 ± 2.0	0.3 ± 0.9	1.0 ± 2.4	0.8 ± 1.9	0.002
TIR (%), mean ± SD	63.8 ± 26.2	66.7 ± 27.4	63.0 ± 26.1	60.2 ± 23.2	0.189
TAR (%), mean ± SD	35.4 ± 26.5	33.4 ± 28.1	35.7 ± 26.2	39.1 ± 23.4	0.323
CV of glucose (%), mean ± SD	24.0 ± 7.4	21.8 ± 5.2	25.1 ± 8.1	24.8 ± 8.2	< 0.001
MAGE (mmol/L), mean ± SD	5.4 ± 2.4	5.1 ± 2.0	5.6 ± 2.6	5.6 ± 2.6	0.099
FT3 (pmol/L), median (IQR)	4.2 (3.7–4.6)	4.3 (3.9–4.7)	4.2 (3.4–4.6)	3.8 (3.3–4.2)	< 0.001
FT4 (pmol/L), median (IQR)	15.8 (14.3–17.7)	15.8 (14.0–17.7)	16.0 (14.5–17.8)	14.9 (13.8–16.8)	0.080
TSH (mIU/L), median (IQR)	2.1 (1.2–3.0)	2.2 (1.3–3.1)	1.9 (1.1–3.0)	2.1 (1.1–3.0)	0.275

Abbreviations: CGMS, continuous glucose monitoring system; CV, coefficient of variation; FT3, triiodothyronine; FT4, thyroxine; HbA1C, hemoglobin A1c; IQR, interquartile range; MAGE, mean amplitude of glycemic excursions; SD, standard deviation; TAR, time above range; TBR, time below range; TIR, time in range; TSH, thyroid stimulating hormone.

### 3.3 Correlations Between CGMS‐Derived Indices and Thyroid Function Indices in All T2DM Patients

FT3 was negatively correlated with HbA1C (*p* < 0.001), mean of glucose (*p* < 0.001), SD of glucose (*p* < 0.001), TAR (*p* < 0.001), and MAGE (*p* = 0.038), whereas it was positively correlated with TIR (*p* < 0.001). FT4 was positively correlated with HbA1C (*p* < 0.001); however, it was not correlated with mean of glucose, SD of glucose, the TBR, the TIR, the TAR, the CV of glucose, or the MAGE. TSH was negatively correlated with HbA1C (*p* = 0.004), the CV of glucose (*p* = 0.019), and MAGE (*p* = 0.027), whereas it was not correlated with mean of glucose, SD of glucose. TBR, TIR, or TAR (Table 3).

**TABLE 3:** Correlation between CGMS‐derived indices and thyroid function indices.

Items	FT3 (pmol/L)		FT4 (pmol/L)		TSH (mIU/L)	
	*p*	*p*	*p*	*p*	*p*	*p*
HbA1C (%)	−0.290	< 0.001	0.185	< 0.001	−0.146	0.004
Mean of glucose (mmol/L)	−0.242	< 0.001	−0.017	0.728	−0.051	0.307
SD of glucose (mmol/L)	−0.174	< 0.001	0.037	0.460	−0.097	0.052
TBR (%)	0.019	0.702	0.012	0.818	−0.004	0.932
TIR (%)	0.237	< 0.001	0.024	0.629	0.062	0.220
TAR (%)	−0.250	< 0.001	−0.024	0.628	−0.052	0.297
CV of glucose (%)	−0.083	0.098	0.055	0.272	−0.117	0.019
MAGE (mmol/L)	−0.104	0.038	0.003	0.946	−0.110	0.027

Abbreviations: CV, coefficient of variation; FT3, triiodothyronine; FT4, thyroxine; HbA1C, hemoglobin A1c; MAGE, mean amplitude of glycemic excursions; SD, standard deviation; TAR, time above range; TBR, time below range; TIR, time in range; TSH, thyroid stimulating hormone.

### 3.4 Associations of CGMS‐Derived Indices and Thyroid Function Indices With Albuminuria

Univariate logistic regression analysis revealed that HbA1C, the SD of glucose, the TBR, the CV of glucose, and MAGE were positively associated with the risk of albuminuria (all *p* < 0.05). Among the basic characteristics, BMI, history of hypertension, SBP, CHO, TG, uric acid, BUN, and Cr were positively associated with the risk of albuminuria, whereas HDL‐C and eGFR were negatively correlated with the risk of albuminuria (all *p* < 0.05). Among medications, benzoic acid derivative use was negatively associated with the risk of albuminuria, whereas CCB and ARB use were positively associated with the risk of albuminuria (all *p* < 0.05). Regarding the risk of macroalbuminuria, FT3 was negatively associated with it (*p* = 0.003). Among the basic characteristics, sex (male vs. female), BMI, history of hypertension, duration of T2DM, SBP, DBP, CHO, LDL‐C, TG, uric acid, BUN, and Cr were positively associated with the risk of macroalbuminuria, whereas the eGFR was negatively associated with the risk of macroalbuminuria (all *p* < 0.05). Among medications, biguanide use was negatively associated with the risk of macroalbuminuria, but CCB and ARB use was positively associated with macroalbuminuria (all *p* < 0.05) (Table 4). On the basis of the ROC analysis, the cutoff values of HbA1C, SD of glucose, the TBR, the CV of glucose, MAGE, and FT3 for the risk of albuminuria and macroalbuminuria were determined and are shown in Table S1.

**TABLE 4:** Univariate logistic regression analysis.

Items	Risk of albuminuria (microalbuminuria and macroalbuminuria vs. normal)		Risk of macroalbuminuria (macroalbuminuria vs. microalbuminuria and normal)	
	OR (95% CI)	*p*	OR (95% CI)	*p*
HbA1C (per %)	1.146 (1.050–1.250)	0.002	1.039 (0.932–1.159)	0.487
Mean of glucose (per mmol/L)	1.059 (0.955–1.174)	0.279	1.050 (0.921–1.197)	0.464
SD of glucose (per mmol/L)	1.670 (1.299–2.147)	< 0.001	1.163 (0.873–1.549)	0.303
TBR (per %)	1.313 (1.102–1.564)	0.002	1.002 (0.881–1.140)	0.970
TIR (per %)	0.994 (0.986–1.001)	0.099	0.994 (0.984–1.004)	0.213
TAR (per %)	1.005 (0.997–1.012)	0.238	1.006 (0.997–1.016)	0.213
CV of glucose (per %)	1.067 (1.036–1.099)	< 0.001	1.019 (0.985–1.054)	0.281
MAGE (per mmol/L)	1.096 (1.007–1.193)	0.033	1.044 (0.943–1.155)	0.410
FT3 (per pmol/L)	0.966 (0.875–1.066)	0.491	0.590 (0.416–0.837)	0.003
FT4 (per pmol/L)	0.990 (0.930–1.054)	0.749	0.923 (0.836–1.019)	0.112
TSH (per mIU/L)	0.981 (0.926–1.039)	0.509	0.941 (0.815–1.088)	0.412
Age (per year)	0.999 (0.986–1.012)	0.891	1.008 (0.990–1.026)	0.390
Sex (male vs. female)	1.049 (0.697–1.578)	0.820	1.938 (1.067–3.519)	0.030
BMI (per kg/m^2^)	1.072 (1.015–1.133)	0.013	1.105 (1.034–1.181)	0.003
Smoke (yes vs. no)	1.302 (0.829–2.044)	0.252	1.587 (0.922–2.733)	0.096
History of hypertension (yes vs. no)	1.819 (1.225–2.701)	0.003	3.619 (1.974–6.636)	< 0.001
Duration of T2DM (per year)	0.991 (0.967–1.015)	0.446	1.052 (1.020–1.085)	0.001
SBP (per mmHg)	1.013 (1.001–1.024)	0.033	1.032 (1.018–1.047)	< 0.001
DBP (per mmHg)	1.017 (0.998–1.036)	0.081	1.024 (1.000–1.047)	0.046
CHO (per mmol/L)	1.213 (1.024–1.436)	0.025	1.322 (1.112–1.571)	0.002
HDL‐C (per mmol/L)	0.407 (0.218–0.760)	0.005	0.721 (0.305–1.708)	0.458
LDL‐C (per mmol/L)	1.116 (0.886–1.405)	0.351	1.368 (1.023–1.830)	0.035
TG (per mmol/L)	1.186 (1.037–1.356)	0.013	1.091 (1.021–1.166)	0.010
Uric acid (per μmol/L)	1.003 (1.001–1.005)	0.001	1.003 (1.001–1.005)	0.001
BUN (per mmol/L)	1.111 (1.028–1.200)	0.008	1.180 (1.096–1.270)	< 0.001
Cr (per μmol/L)	1.010 (1.004–1.017)	0.002	1.017 (1.011–1.023)	< 0.001
eGFR (per mL/min/1.73 m^2^)	0.992 (0.987–0.998)	0.005	0.969 (0.959–0.979)	< 0.001
Insulin use (yes vs. no)	0.919 (0.558–1.513)	0.739	1.490 (0.728–3.046)	0.275
Sulfonylureas use (yes vs. no)	1.375 (0.796–2.376)	0.254	0.432 (0.180–1.038)	0.061
Benzoic acid derivative use (yes vs. no)	0.546 (0.307–0.970)	0.039	0.531 (0.204–1.384)	0.195
Biguanide use (yes vs. no)	0.947 (0.627–1.430)	0.795	0.549 (0.327–0.923)	0.024
DPP‐4 inhibitor use (yes vs. no)	0.971 (0.623–1.515)	0.897	0.922 (0.509–1.668)	0.788
Glycosidase inhibitor use (yes vs. no)	0.895 (0.600–1.333)	0.584	1.080 (0.640–1.821)	0.774
TZD use (yes vs. no)	0.760 (0.126–4.598)	0.765	1.390 (0.153–12.623)	0.770
GLP‐1 inhibitor use (yes vs. no)	2.317 (0.933–5.758)	0.070	2.773 (1.250–6.151)	0.012
SGLT‐2 inhibitor use (yes vs. no)	0.573 (0.258–1.272)	0.171	0.709 (0.207–2.431)	0.585
β‐receptor blocker use (yes vs. no)	1.170 (0.559–2.446)	0.677	1.117 (0.447–2.795)	0.812
CCB use (yes vs. no)	1.925 (1.178–3.146)	0.009	3.080 (1.804–5.256)	< 0.001
ACE inhibitor use (yes vs. no)	0.912 (0.300–2.772)	0.872	0.920 (0.201–4.206)	0.915
ARB use (yes vs. no)	1.962 (1.253–3.073)	0.003	3.786 (2.231–6.427)	< 0.001
MRA use (yes vs. no)	2.328 (0.497–10.910)	0.284	2.125 (0.550–8.217)	0.275
Diuretic use (yes vs. no)	1.995 (0.730–5.451)	0.178	2.424 (0.966–6.086)	0.059
α‐receptor blocker use (yes vs. no)	0.505 (0.070–3.621)	0.497	1.858 (0.190–18.124)	0.594

Abbreviations: ACE, angiotensin‐converting enzyme; ARB, angiotensin II receptor blocker; BMI, body mass index; BUN, blood urea nitrogen; CCB, calcium channel blocker; CHO, cholesterol; CI, confidence interval; Cr, creatinine; CV, coefficient of variation; DBP, diastolic blood pressure; DPP‐4, dipeptidyl peptidase‐4; eGFR, estimated glomerular filtration rate; FT3, triiodothyronine; FT4, thyroxine; GLP‐1, glucagon‐like peptide‐1; HbA1C, hemoglobin A1c; HDL‐C, high‐density lipoprotein cholesterol; IQR, interquartile range; IQR, interquartile range; LDL‐C, low‐density lipoprotein cholesterol; MAGE, mean amplitude of glycemic excursions; MRA, mineralocorticoid receptor antagonist; OR, odds ratio; SBP, systolic blood pressure; SD, standard deviation; SGLT‐2, sodium–glucose cotransporter 2; T2DM, type 2 diabetes mellitus; TAR, time above range; TBR, time below range; TG, triglyceride; TIR, time in range; TSH, thyroid stimulating hormone; TZD, thiazolidinedione.

Considering the interference of basic characteristics, the associations of CGMS‐derived indices and thyroid function indices with albuminuria and macroalbuminuria were adjusted by Model 1 (demographics), Model 2 (demographics and disease‐related indices), Model 3 (demographics, disease‐related indices, and biochemical indices), and Model 4 (demographics, disease‐related indices, biochemical indices, and medication information). The results revealed that HbA1C, the SD of glucose, the TBR, the CV of glucose, and MAGE were independently associated with a greater risk of albuminuria after adjustment for Model 1, Model 2, Model 3, and Model 4 (all *p* < 0.05). However, FT3 was not independently associated with the risk of albuminuria after adjustment for the three models (all *p* > 0.05). In terms of the risk of macroalbuminuria, HbA1C (*p* < 0.05), the SD of glucose (*p* = 0.041), the CV of glucose (*p* = 0.046), and MAGE (*p* = 0.040) were independently associated with a greater risk of macroalbuminuria after adjustment for Model 2. The CV of glucose was independently associated with a greater risk of macroalbuminuria after adjustment for Model 4 (*p* = 0.044). FT3 was independently associated with a lower risk of macroalbuminuria after adjustment for Model 1 (*p* < 0.001), Model 2 (*p* < 0.001), and Model 4 (*p* = 0.009) (Table 5).

**TABLE 5:** Multivariate logistic regression analysis.

Items	Risk of albuminuria (microalbuminuria and macroalbuminuria vs. normal)		Risk of macroalbuminuria (macroalbuminuria vs. microalbuminuria and normal)	
	OR (95% CI)	*p*	OR (95% CI)	*p*
HbA1C (per %)				
Model 1	1.199 (1.088–1.321)	< 0.001	1.082 (0.964–1.215)	0.182
Model 2	1.186 (1.076–1.308)	0.001	1.128 (1.000–1.273)	0.050
Model 3	1.197 (1.076–1.333)	0.001	1.070 (0.933–1.226)	0.333
Model 4	1.244 (1.109–1.396)	< 0.001	1.095 (0.933–1.286)	0.266
SD of glucose (per mmol/L)				
Model 1	2.039 (1.533–2.710)	< 0.001	1.367 (0.993–1.882)	0.056
Model 2	2.032 (1.525–2.709)	< 0.001	1.406 (1.013–1.951)	0.041
Model 3	2.233 (1.635–3.051)	< 0.001	1.278 (0.869–1.879)	0.213
Model 4	2.404 (1.734–3.33)	< 0.001	1.341 (0.886–2.030)	0.166
TBR (per %)				
Model 1	1.345 (1.117–1.621)	0.002	1.032 (0.907–1.175)	0.630
Model 2	1.347 (1.118–1.622)	0.002	1.029 (0.904–1.172)	0.664
Model 3	1.401 (1.158–1.695)	0.001	1.047 (0.897–1.223)	0.560
Model 4	1.399 (1.155–1.696)	< 0.001	1.045 (0.881–1.239)	0.615
CV of glucose (per %)				
Model 1	1.090 (1.054–1.127)	< 0.001	1.037 (0.999–1.075)	0.056
Model 2	1.089 (1.053–1.126)	< 0.001	1.039 (1.001–1.078)	0.046
Model 3	1.114 (1.073–1.157)	< 0.001	1.039 (0.993–1.087)	0.098
Model 4	1.122 (1.079–1.167)	< 0.001	1.052 (1.001–1.105)	0.046
MAGE (per mmol/L)				
Model 1	1.160 (1.055–1.275)	0.002	1.116 (0.997–1.249)	0.057
Model 2	1.157 (1.052–1.272)	0.003	1.128 (1.005–1.266)	0.040
Model 3	1.188 (1.073–1.315)	0.001	1.121 (0.979–1.283)	0.097
Model 4	1.213 (1.090–1.350)	< 0.001	1.165 (1.004–1.351)	0.044
FT3 (per pmol/L)				
Model 1	0.964 (0.869–1.070)	0.495	0.468 (0.315–0.694)	< 0.001
Model 2	0.961 (0.866–1.066)	0.451	0.451 (0.298–0.681)	< 0.001
Model 3	0.982 (0.854–1.129)	0.799	0.645 (0.391–1.063)	0.086
Model 4	1.002 (0.872–1.152)	0.977	0.463 (0.260–0.826)	0.009

*Note:* Model 1, adjusted for demographics; Model 2, adjusted for demographics and disease‐related indices; Model 3, adjusted for demographics, disease‐related indices, and biochemical indices; Model 4, adjusted for demographics, disease‐related indices, biochemical indices, and medication information.

### 3.5 Mediation Analyses

Mediation analyses were performed to test whether the association of glucose variability with the risk of albuminuria was mediated by thyroid function indices. The data revealed that the associations of HbA1C, the SD of glucose, the TBR, the CV of glucose, and MAGE with the risk of albuminuria were partially mediated by FT3, FT4, and TSH. However, the weight of mediation was generally low, ranging from −6.320% to 9.982% (Table 6).

**TABLE 6:** Mediation analyses of the risk of albuminuria.

Independent variable	Mediating variable	Dependent variable	Total effect (95% CI), *p*	Indirect effect (95% CI), *p*	Direct effect (95% CI), *p*	Mediation
HbA1C	FT3	Risk of albuminuria	0.027 (0.006–0.038), 0.014	0.001 (−0.001–0.010), 0.536	0.027 (0.004–0.037), 0.024	1.895%
SD of glucose	FT3	Risk of albuminuria	0.116 (0.057–0.166), < 0.001	−0.001 (−0.005–0.029), 0.802	0.117 (0.051–0.167), < 0.001	−1.124%
TBR	FT3	Risk of albuminuria	0.059 (0.026–0.109), < 0.001	−0.004 (−0.012–0.001), 0.156	0.063 (0.029–0.117), < 0.001	−6.320%
CV of glucose	FT3	Risk of albuminuria	0.013 (0.008–0.015), < 0.001	0.000 (−0.001–0.001), 0.442	0.014 (0.008–0.016), < 0.001	−2.821%
MAGE	FT3	Risk of albuminuria	0.020 (0.001–0.043), 0.036	−0.001 (−0.003–0.008), 0.546	0.021 (0.000–0.043), 0.056	−5.602%
HbA1C	FT4	Risk of albuminuria	0.027 (0.005–0.037), 0.010	0.001 (−0.001–0.004), 0.244	0.026 (0.005–0.037), 0.010	2.699%
SD of glucose	FT4	Risk of albuminuria	0.116 (0.059–0.168), < 0.001	0.004 (−0.004–0.026), 0.428	0.113 (0.055–0.164), < 0.001	3.180%
TBR	FT4	Risk of albuminuria	0.058 (0.028–0.107), < 0.001	0.002 (−0.003–0.014), 0.524	0.056 (0.025–0.106), < 0.001	3.338%
CV of glucose	FT4	Risk of albuminuria	0.013 (0.009–0.016), < 0.001	0.000 (0.000–0.003), 0.468	0.013 (0.008–0.015), < 0.001	2.942%
MAGE	FT4	Risk of albuminuria	0.021 (0.002–0.044), 0.034	0.002 (−0.001–0.012), 0.312	0.019 (−0.002–0.040), 0.068	9.982%
HbA1C	TSH	Risk of albuminuria	0.027 (0.006–0.038), 0.010	0.000 (−0.003–0.004), 0.748	0.027 (0.006–0.037), 0.012	1.690%
SD of glucose	TSH	Risk of albuminuria	0.117 (0.060–0.168), < 0.001	0.001 (−0.006–0.007), 0.818	0.116 (0.059–0.168), < 0.001	0.653%
TBR	TSH	Risk of albuminuria	0.057 (0.030–0.109), < 0.001	0.000 (−0.001–0.001), 0.834	0.057 (0.030–0.108), < 0.001	0.113%
CV of glucose	TSH	Risk of albuminuria	0.013 (0.009–0.015), < 0.001	0.000 (−0.001–0.000), 0.766	0.013 (0.009–0.016), < 0.001	0.591%
MAGE	TSH	Risk of albuminuria	0.020 (0.000–0.040), 0.054	0.000 (−0.002–0.002), 0.770	0.020 (−0.001–0.040), 0.058	1.466%

Abbreviations: CI, confidence interval; CV, coefficient of variation; FT3, triiodothyronine; FT4, thyroxine; HbA1C, hemoglobin A1c; MAGE, mean amplitude of glycemic excursions; SD, standard deviation; TBR, time below range; TSH, thyroid stimulating hormone.

## 4 Discussion

This study enrolled patients with T2DM and detected the indices generated by the CGMS and the levels of thyroid hormones. According to the UACR ratio, 299 out of 451 patients were identified with albuminuria, accounting for 66.3%, and 69 out of 451 patients were identified with macroalbuminuria, accounting for 15.3%. Compared with two previous studies conducted in Japan, the prevalence rates of albuminuria and macroalbuminuria were greater in the present study [11, 12]. A possible explanation was that this study included mainly Chinese patients, and the differences in dietary habits and control of T2DM might have resulted in this discrepancy.

In this study, several indices generated by the CGMS were associated with the level of albuminuria. Specifically, HbA1C, the SD of glucose, TBR, MAGE, and the CV of glucose were all positively associated with the level of albuminuria. These positive associations survived after adjustment for patient demographics, disease‐related features, and biochemical indices. HbA1C reflects the level of blood glucose 2–3 months prior to investigation [18], and a higher level of HbA1C was associated with inadequate control of blood glucose over this period. The positive association of HbA1C with albuminuria in patients with T2DM was in accordance with previous studies [19, 20, 21], which highlighted the importance of adequate blood control for preventing the progression of diabetic nephropathy. The SD and CV of blood glucose and MAGE reflected the variability in blood glucose during the study period. The positive associations of the SD and CV of blood glucose and MAGE with albuminuria suggested that a greater fluctuation in blood glucose was associated with the progression of diabetic nephropathy. These findings are in line with those of previous studies [11, 19], which emphasized that stable control of blood glucose is critical for preventing diabetic nephropathy. However, the effect sizes of the associations of HbA1C, the CV of glucose, and MAGE with albuminuria were not large, suggesting that the contributions of these factors to albuminuria might lack clinical significance. The positive association of TBR with albuminuria contradicted the findings of a previous study [11]. The TBR reflects the occurrence of hypoglycemia, suggesting the instability of glycemic control, which increases the risk of microvascular damage, leading to the occurrence of diabetic nephropathy. In addition, it has also been reported that decreased renal function increases the risk of hypoglycemia, resulting in a positive association between TBR and the UACR [22]. Nevertheless, the TBR was very low overall in patients with T2DM, suggesting that most patients did not experience hypoglycemia. Therefore, the TBR might be a marker of intensive therapy or insulin use rather than a causal factor.

The thyroid is a vital endocrine gland that regulates energy metabolism [23]. The current study also investigated the associations of thyroid hormones with albuminuria in patients with T2DM. The data revealed that FT3 was negatively associated with macroalbuminuria in these patients, which was in accordance with the findings of previous studies [14, 24, 25]. However, the current study revealed that FT4 or TSH was not associated with albuminuria in patients with T2DM. After adjustment for demographics or demographics plus disease characteristics, FT3 was still negatively associated with macroalbuminuria in these patients. However, the association between FT3 and macroalbuminuria diminished after adjustment for biochemical indices, including renal function. Therefore, lower FT3 might reflect poor renal status or systemic illness rather than being an independent causal factor. These findings also highlight that diabetic nephropathy is a systemic issue, which reinforces the demand for systemic management of this disease. However, FT4 and TSH were not associated with macroalbuminuria. In addition, FT3, FT4, and TSH were generally within the normal range, suggesting that lower FT3 might reflect nonthyroidal illness syndrome rather than true thyroid dysfunction.

The results of the mediation analyses revealed that the association of glucose variability with the risk of albuminuria was partially mediated by thyroid function, although the weight of mediation was not high. Therefore, the influence of poor glucose control on worsening renal function in patients with T2DM was mainly through direct effects, with a small proportion mediated by thyroid function. These findings suggest that poor glucose control might directly induce renal dysfunction. The mechanisms involved in this process might include oxidative stress, inflammation, and advanced glycation end product‐mediated injury [26, 27, 28]. However, the involvement of endocrine regulation in this process might not be significant. Notably, the thyroid function indices were generally within the normal range, and whether similar findings could be observed in patients with thyroid dysfunction should be further explored. Importantly, given the cross‐sectional design of this study, the mediation analysis cannot establish temporal or causal relationships among glycemic variability, thyroid hormones, and albuminuria. Therefore, the observed mediation effects should be interpreted with caution and considered exploratory in nature.

This study provided the cutoff values of HbA1C, the SD of glucose, the TBR, the CV of glucose, MAGE, and FT3 for the risk of albuminuria and macroalbuminuria, which could support the prediction of diabetic renal disease in patients with T2DM. Moreover, clinicians could stratify patients with a greater risk of renal dysfunction on the basis of these data, thus providing additional management to reduce the incidence of renal dysfunction. However, these cutoff values were derived from ROC analysis, and further verification is needed. Although the logistic regression analysis figured out the association of HbA1C, the SD of glucose, the TBR, the CV of glucose, MAGE, and FT3 with albuminuria or macroalbuminuria, the direction of causality could not be determined. It is also likely that T2DM patients with renal dysfunction have worse glucose control and lower FT3. Therefore, further prospective studies are needed to explore the direction of causality between glucose variability and thyroid function indices and renal function in patients with T2DM.

The correlations between indices generated by the CGMS and thyroid hormones in patients with T2DM are not clear. The correlation analyses in this study revealed that FT3 was correlated with most of the indices generated by the CGMS; generally, FT3 was negatively correlated with HbA1C, glucose, and variability of glucose. However, FT4 and TSH did not show strong correlations with indices generated by CGMS, and statistical significance was not reached in most of the correlation analyses. These findings suggest that there is crosstalk among glucose variability, thyroid hormones, and diabetic nephropathy. Previous studies have shown that both glucose fluctuations and insufficient FT3 levels lead to the pathogenesis and progression of diabetic nephropathy [29, 30, 31, 32, 33]. Moreover, glucose fluctuations and FT3 might be negatively regulated by each other, thus further contributing to diabetic nephropathy. However, the causal relationship between glucose variability and FT3 should be investigated in further studies.

There were several limitations to this study. First, the number of patients with macroalbuminuria was not large enough, which could lead to low statistical power in the relevant analyses. Second, due to the cross‐sectional design, the temporal sequence among glucose variability, thyroid function, and albuminuria could not be established. Accordingly, the mediation analysis performed in this study does not support causal inference and should be interpreted as exploratory. Third, most of the patients were from China, which led to a lack of generalizability of our findings. Fourth, this study included only T2DM patients who wore CGMS. In our hospital, CGMS is prescribed for hospitalized patients with inadequate glycemic control or marked glycemic variability. Therefore, including only T2DM patients who wore CGMS would hamper the external validity, reduce the generalizability, and potentially increase the observed prevalence of albuminuria. In addition, the pathophysiology of renal dysfunction and the degree of glycemic variability in patients with T2DM could be fully understood only in these patients. Fifth, this study used a single value of UACR rather than a mean value of repeated measurements of UACR, which could induce potential bias.

In conclusion, glycemia and glucose variability derived from the CGMS are correlated with lower FT3, and they are positively correlated with albuminuria in patients with T2DM. In addition, the associations of glycemia and glucose variability with albuminuria are mainly direct effects, with a small proportion mediated by thyroid function.

## Funding

This study was supported by the Northern Jiangsu People's Hospital support technology project (No. FCJS202337).

## Conflicts of Interest

The authors declare no conflicts of interest.

## Supporting information


**Table S1:** The cutoff values of CGMS‐derived indices or thyroid function indices with *p* < 0.05 in univariate analyses.

## Data Availability

The datasets generated during and/or analyzed during the current study are available from the corresponding author on reasonable request.
