# Supplementary material for: Correlations Among Glycemia and Glucose Variability, Thyroid Hormones, and Albuminuria in Patients With Type 2 Diabetes Mellitus
Source: Kaohsiung J Med Sci. 2026 Apr 22:e70221. Online ahead of print. doi: 10.1002/kjm2.70221 (PMC13399747; doi:10.1002/kjm2.70221)
Supplement: Supplementary file 1 — Table S1: The cutoff values of CGMS‐derived indices or thyroid function indices with p < 0.05 in univariate analyses. [file KJM2-9999-e70221-s001.docx]

**Supplementary Table 1.** The cutoff values of CGMS-derived indices or thyroid function indices with *P* <0.05 in univariate analyses.

| Items | Cutoff values | |
| --- | --- | --- |
|  | Risk of albuminuria  (microalbuminuria and macroalbuminuria vs. normal) | Risk of macroalbuminuria  (macroalbuminuria vs. microalbuminuria and normal) |
| HbA1C (%) | 11.0 | 12.3 |
| SD of glucose (mmol/L) | 2.6 | 1.7 |
| TBR (%) | 0.5 | 2.5 |
| CV of glucose (%) | 29.8 | 26.2 |
| MAGE (mmol/L) | 8.4 | 5.7 |
| FT3 (pmol/L) | 5.2 | 2.6 |

CGMS, continuous glucose monitoring system; HbA1c, hemoglobin A1c; SD, standard deviation; TBR, time below range; CV, coefficient of variation; MAGE, mean amplitude of glycemic excursions; FT3, triiodothyronine.
